# A systematic review of the economic impact of rapid diagnostic tests for dengue

**DOI:** 10.1186/s12913-017-2789-8

**Published:** 2017-12-29

**Authors:** Jacqueline Kyungah Lim, Neal Alexander, Gian Luca Di Tanna

**Affiliations:** 1 Global Dengue and Aedes-transmitted Diseases Consortium (GDAC), International Vaccine Institute (IVI), SNU Research Park, Gwankak-ro 1, Seoul, Gwanak-gu 151-191 South Korea; 2 Epidemiology and Public Health Department, London School of Hygiene and Tropical Medicine, London, UK; 3 Centre for Primary Care and Public health, Queen Mary University of London, London, UK

**Keywords:** Dengue, Dengue fever, Diagnostic, rapid diagnostic test (RDT), Cost-effectiveness

## Abstract

**Background:**

Dengue fever is rapidly expanding geographically, with about half of the world’s population now at risk. Among the various diagnostic options, rapid diagnostic tests (RDTs) are convenient and prompt, but limited in terms of accuracy and availability.

**Methods:**

A systematic review was conducted of published data on the use of RDTs for dengue with respect to their economic impact. The search was conducted with combinations of key search terms, including “((Dengue[Title]) AND cost/economic)” and “rapid diagnostic test/assay (or point-of-care)”. Articles with insufficient report on cost/economic aspect of dengue RDTs, usually on comparison of different RDTs or assessment of novel rapid diagnostic tools, were excluded. This review has been registered in the PROSPERO International prospective register of systematic reviews (registry #: CRD42015017775).

**Results:**

Eleven articles were found through advanced search on Pubmed. From Embase and Web of Science, two and 14 articles were obtained, respectively. After removal of duplicate items, title screening was done on 21 published works and 12 titles, including 2 meeting abstracts, were selected for abstract review. For full-text review, by two independent reviewers, 5 articles and 1 meeting abstract were selected. Among these, the abstract was referring to the same study results as one of the articles. After full text review, two studies (two articles and one abstract) were found to report on cost-wise or economic benefits of dengue RDTs and were selected for data extraction. One study found satisfactory performance of IgM-based Panbio RDT, concluding that it would be cost-effective in endemic settings. The second study was a modeling analysis and showed that a dengue RDT would not be advantageous in terms of cost and effectiveness compared to current practice of antibiotics prescription for acute febrile illness.

**Conclusions:**

Despite growing use of RDTs in research and clinical settings, there were limited data to demonstrate an economic impact. The available two studies reached different conclusions on the cost-effectiveness of dengue RDTs, although only one of the two studies reported outcomes from cost-effectiveness analysis of dengue and the other was considering febrile illness more generally. Evidence of such an impact would require further quantitative economic studies.

**Electronic supplementary material:**

The online version of this article (10.1186/s12913-017-2789-8) contains supplementary material, which is available to authorized users.

## Background

Dengue fever, a mosquito-borne flavivirus infection caused by four related but antigenically distinct dengue viruses (DENVs, serotypes 1–4), is a major and rapidly increasing public health problem. Its geographic range now includes about half of the world’s population and continues to expand, with epidemics that disrupt health care systems [1–4]. Current WHO estimates are of about 50–100 million annual infections globally, while Bhatt et al. recently estimated 390 million infections annually with 96 million disease episodes [5–7].

However, there are no other suitable disease prevention methods: mosquito vector control is often ineffective [8, 9]. There is a vaccine, Sanofi Pasteur’s live attenuated Dengvaxia®, recently registered in multiple countries in Southeast Asia and Latin America and it shows to have variable efficacy [10–13]. At present, there are no drugs for specific treatment and there is a need for accurate and cheap dengue diagnostic tests to be widely used in clinical settings [14–16]. Thus, many dengue endemic countries in the tropics are still experiencing a rise in cases and in deaths due to dengue [17–20].

Recently the WHO Strategic Advisory Group of Experts (SAGE) on Immunization emphasized the need for estimation of the true burden of dengue disease, including cost of illness [6]. Data are available, but mostly focused in countries in Asia and Latin America, with well-documented hyper-endemicity and a long history of dengue transmission, such as Thailand [21, 22], the Philippines [23], Brazil [24, 25], Mexico [26], and Colombia [27]. Most of the available burden data are from studies of the epidemiology and evidence based on economic studies is limited [28, 29].

Among the key limitations of economic studies of dengue are the challenges in its diagnosis. Often, cost-related studies for dengue are based on clinical, rather than laboratory, confirmation [30]. Available methods include virus isolation, serology, and molecular methods [31]. One test routinely used by research laboratories for virus identification is Reverse Transcriptase-Polymerase Chain Reaction (RT-PCR) assay [32]. While this is a definite proof of infection and confirms the serotype, commercial kits that include serotyping are often expensive and would require serum samples collected in early phase during the illness [33]. Another commonly used method is immunoglobulin type M (IgM) antibody capture enzyme-linked immunosorbent assay (MAC-ELISA) [31]. With IgM staying elevated for 2 to 3 months, interpretation could be challenging given that elevated IgM could be due either to recent past infection or to cross-reactivity with other flaviviruses [33]. Any clinical management decision reached on the basis of a single blood sample collected in the acute phase is not conclusive. Levels of immunoglobulin type G (IgG) stay elevated for months to years, so a positive result on one of the available assays could indicate a past infection, thus has limited implications for clinical management [31]. Moreover, it may cross-react across the Flavivirus group (dengue virus, Japanese encephalitis virus, West Nile virus, yellow fever virus, Zika virus, etc.) [34]. There are other assays such as Plaque Reduction Neutralization Test (PRNT) which detect serotype-specific antibodies [35]. Compared to others mentioned, these are time-consuming and labour-intensive, thus expensive [36, 37].

Amongst different diagnostic tools, rapid diagnostic tests (RDTs) are a convenient (easy to use) and prompt option, despite their limitations in terms of accuracy [38]. While their availability could be limited, especially in resource-limited settings, RDTs are commonly used for dengue detection in many endemic countries [38]. There could be a number of different commercially available tests and they could be based on the detection of dengue virus non-structural protein 1 (NS1) antigen, IgM, IgG, and IgA antibodies [39]. Often, these tests have high specificity (usually around 90%), but lower levels of sensitivity, ranging from 10 to 99%, in detection of dengue and could be cross-reactive with other flaviviruses [39–42]. However, the speed of RDTs provides early diagnosis of dengue possibly leading to timely case management. Given their limited accuracy, these RDTs are not considered the standard reference and their usefulness is not yet proven in clinical settings [15, 43]. However, some literature supports the use of such tests in combination with others, for example the combined test with NS1 antigen and IgM antibody [42, 44].

Especially in terms of economic studies, one major benefit of using RDTs would be that they allow dengue detection in the early phase of illness (at presentation), hence facilitating capture of the entire spectrum of costs incurred throughout illness. Previous studies reported that early detection is effective in reducing the duration of illness, possibly leading to lower cost-of-illness due to dengue [45, 46]. In recognition of the need to balance speed, accuracy, and availability to maximize utility when using RDTs for dengue detection for the patients in clinical settings, a systematic review was performed to explore the economic impact of using RDTs for dengue. The hypothesis behind this review was that there may be economic impact due to prompt detection of dengue in the early phase of illness using RDTs and economic impact is defined to be broad: both from the point of view of cost-effectiveness and from the perspective of financial impact of RDT in patients, i.e. early diagnosis possibly leading to cost-saving in patients.

## Methods

In this review, literature published in English up to September 2017 was covered. Scientific databases used for the search were: Embase, IBSS, Medline (including PubMed), and Web of Science. In order to take more caution and not miss articles that may imply on economic benefit of RDTs, the literature search was conducted in a comprehensive approach. In Pubmed, advance search was performed with search terms “((Dengue[Title]) AND cost)” OR “((Dengue[Title]) AND economic)” AND:

1. “rapid diagnostic test[MeSH Terms]”
2. “RDT[MeSH Terms]”
3. “rapid test[MeSH Terms]”
4. “rapid assay[MeSH Terms]”
5. “rapid diagnostic assay[MeSH Terms]”
6. “point-of-care [MeSH Terms]”
7. “POC[MeSH Terms]”
8. “point-of-care test[MeSH Terms]”

MeSH terms are assigned by indexers of the National Library of Medicine [47]. While the search on Pubmed was performed with above search terms with “rapid diagnostic test” and “point-of-care test” were used as MeSH terms, additional articles were identified through IBSS, EMBASE, and Web of Science via general search using keywords:

1. “dengue and rapid diagnostic test (or RDT) and cost”
2. “dengue and rapid diagnostic test (or RDT) and economic”
3. “dengue and point-of-care (or POC) and cost”
4. “dengue and point-of-care (or POC) and economic”.

From Embase, Web of Science, and WHOLIS, outcomes of general search included meeting abstracts in addition to full articles. Preliminary screening needed to be done for search outcomes through Embase and Web of Science, as their general search led to journals, not articles, where each key word may appear in different articles.

**Fig. 1:**
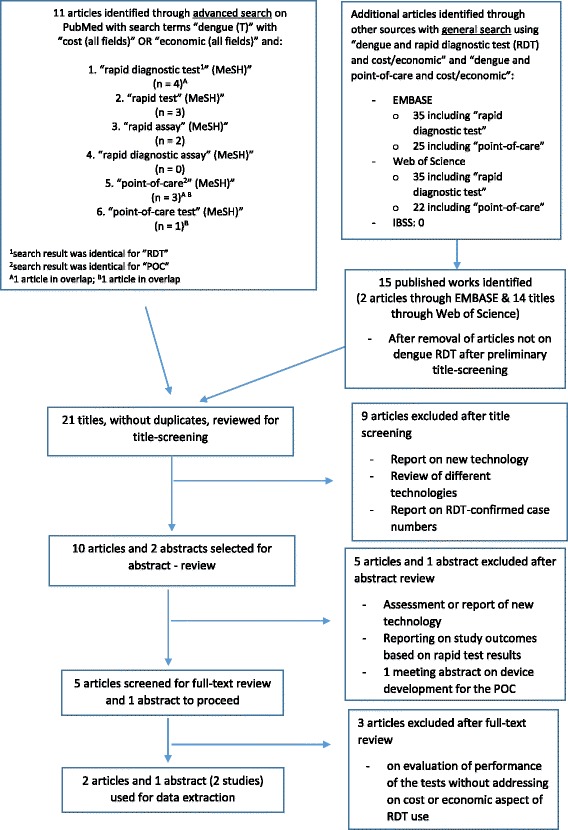
Flow of the literature search in the systematic review

After such preliminary screening was done for search outcomes through Embase and Web of Science, title screening, abstract review, and full-text review were done. The development of this literature review is shown in the flow chart (Fig. 1). Rationales for excluding articles obtained through this multiple searches using different sources were described in Fig. 1. Exclusion criteria were not relevant articles that:

- mainly report on cost associated with a new diagnostic technology
- report on different technologies or performance of the tests without addressing cost or economic aspect of RDT use
- report on RDT-confirmed dengue case numbers in a study with insufficient information on economic impact

Also included for full-text review were those describing, in addition to those with direct reporting of quantitative costs, some qualitative economic benefit, i.e. mention of cost-effectiveness of RDTs without any quantitative valuation of it. This was done to prevent loss of any articles containing cost-related implication, even if not quantitatively specified in the article. Data were then extracted from the full texts of the selected articles. The data extraction table was developed following the Consolidated Health Economic Evaluation Reporting Standards (CHEERS) statement and the reporting Checklist [48]. Also, the reporting Checklist for Cost-effectiveness Analyses from Second Panel on Cost-Effectiveness in Health and Medicine (Additional file 1: Table S1) [48, 49]. In addition, the Preferred Reporting Items for Systematic Reviews and Meta-Analyses (PRISMA) were followed [48, 50]. This review is registered in the PROSPERO international prospective register of systematic reviews, under the title “Systematic review of health economic assessments of dengue rapid diagnostic tests” (registry number: CRD42015017775). Full text review was performed independently by two individuals.

## Results

As shown in Fig. 1, a more focused advanced search on Pubmed resulted in 11 articles and, after preliminary screening of the outcomes of general search on EMBASE and Web of Science, there were 15 published works (2 articles from EMBASE and 14 titles from Web of Science with one in overlap). After removal of duplicative articles, 21 titles from all three sources underwent screening. For abstract review, 10 articles and 2 meeting abstracts were considered relevant and among these 5 articles were selected for full-text review and 1 meeting abstract was retained and, as an abstract, skipped the full-text review step.

On review of the full text of the articles, it was found that only two studies reported quantitative or qualitative economic impact of RDT use for dengue: one by Lubell et al. and another by Mitra et al. The second study was reported in an abstract and an article, the former being published first [51, 52]. As the abstract was referring to the same study results as the articles, they were merged in the data extraction stage and were presented as one combined piece of work. Some others found with “cost” or “economic” as one of the key search terms and reached the full-text review stage were proven to contain information on the cost aspects of dengue RDTs. However, some were found to report on the actual price of the test or cost of production of a new assay as they assess performance of it. The extracted data and findings from these two studies are included in Additional file 1: Tables S1, 2a, and 2b [51–53].

The article (2016) and meeting abstract (2014) by Mitra et al. report that Panbio RDT alone is highly sensitive and cost effective for diagnosis of dengue infection in their comparative evaluation of performance and cost-effectiveness of commercially available immunochromatography-based RDT kits [51, 52]. This study is not, in fact, a cost-effectiveness analysis and reports a high sensitivity (97%) for Panbio RDT and a cost of 13.6 USD (reported in the abstract in 2014, 6.90 USD in the article in 2016) which they refer to as being cost-effective without clearly defining the basis of measurements, i.e. denominator [51, 52]. They compared four commercially available RDTs [Panbio Dengue Duo cassette, Standard Diagnostics (SD) Bioline Dengue Duo, J. Mitra Dengue Day-1 test and Reckon Dengue IgG/IgM] against composite reference criteria (CRC), and compared the cost of the tests. The authors conducted this study among stored blood samples from 281 patients who sought care for acute febrile illness at Christian Medical College (CMC) hospital in Vellore, India [52]. The CRC was locally developed by infectious diseases expert, virologist, epidemiologist, reflecting WHO guidelines and this was used to identify dengue cases while lab-confirmed etiology of other cases of fever was needed to identify non-dengue controls. The authors measured sensitivity, specificity, and predictive values of these commercial RDTs in dengue cases and non-dengue controls. Based on IgM capture positivity of the four selected RDT kits, Panbio test was found to have the highest sensitivity, followed by SD Duo (97.7 and 64.3% respectively) [52]. However, specificity was higher for the Reckon RDT and SD Duo at 99.3 and 96.6%, respectively, compared to Panbio at 87.8%. Based on NS1 antigen capture assay, none were found to show satisfactory results in terms of sensitivity, while specificity was high, around 90% [52]. Therefore, even though the cost of Panbio test was the highest at 6.90 USD (in 2016) compared to the rest three ranging between 3.29 to 4.27 USD, it was concluded that IgM assay by Panbio would be the test of choice and a cost-effective option for diagnosis of acute dengue infection in endemic settings.

An economic evaluation based on cost-effectiveness modeling by Lubell et al. (2016) reported that use of a dengue RDT is found to be not advantageous, more costly and less effective, when compared to the common practice of presumptive treatment with antibiotics prescription [53]. The authors developed a model to measure the impact and cost-effectiveness of testing for elevated C-reactive protein (CRP), compared with RDTs for dengue and scrub typhus in the management of undifferentiated fever. They used data from 1083 outpatients between 5 and 49 years of age from three provincial hospitals in rural Laos [53]. A decision tree model was developed to determine cost effectiveness of different testing approaches for undifferentiated fever and measure the ability of dengue and scrub typhus rapid tests, compared with testing for elevated CRP, to inform antibiotic treatment as currently practiced in clinical settings. The authors assumed sensitivity and specificity of a dengue RDT to be 95% and conducted economic evaluation to calculate the median incremental cost, the number of disability adjusted life years (DALYs) averted, and incremental cost-effectiveness ratios (ICER) for each strategy compared to the current practice of antibiotics prescription. For this, the model adopted assumptions in sensitivity and specificity of tests, costs of tests, the cost of a course of antibiotic, duration of all self-limiting viral infections and treated bacterial infection, as well as duration of bacterial infections that do not receive an appropriate treatment, mortality rate, a mean loss of life-years for a case of death, etc. Another important parameter in the model was incidence. The authors used incidence estimates of different pathogens to calculate proportion of patients who were given antibiotics for bacterial infections and proportion of those given antibiotics for viral infections. Furthermore, variable level of incidence between half to double of what was found in the fever study was applied in the model to test robustness of model outcomes. The model output reported that a dengue RDT is dominated by current practice, with a higher cost (median incremental cost = $1.5, Crl: 0.5; 3.2) and fewer numbers of DALYs averted (−0.006 DALYs, CrI: −0.301; 0.089) on average.

## Discussion

The hypothesis behind this review was that prompt detection of dengue in the early phase of illness using RDTs may lead to economic benefit in terms of patients’ cost of illness. The review was from both the point of view of cost effectiveness of RDT and the perspective of financial impact of RDT. We found two studies with different conclusions [51–53]. Two studies were heterogenous in terms of design — cost-effectiveness modelling or comparative evaluation of performance of RDTs. They both took place in dengue-endemic locations, in India and in Laos, over different time periods between 2008 and 2013 [51–53]. In both studies, the authors acknowledged limited generalizability to other populations of febrile patients, possibly due to specific epidemiological characteristics of each study area [51–53]. Epidemiological profiles, such as a varying level of sero-prevalence and likely high proportion of secondary infections [42], and particular serotype profiles [54, 55] could affect performance of RDTs for detection of dengue.

In the comparative evaluation of performance of RDTs, the authors concluded that Panbio RDT is cost-effective. Performance of IgM assay by Panbio was the most satisfactory in the diagnosis of acute dengue infection and the cost of the test was acceptable. This was although the cost based on the manufacturer’s quoted price in India for Panbio was the highest at 6.90 USD compared to the rest three: SD, Reckon and J. Mitra at US$ 4.27, 3.29 and 3.61, respectively. The authors also explored different combinations. When NS1 antigen capture positivity alone was considered, all three tests (Panbio is IgM assay only) showed sensitivity below 30% while specificity was satisfactory, higher than 90% for all three tests. Thus, the authors concluded the NS1-based test to be unreliable. Also, the authors explored changes in performance when combined tests were used. Paired with Panbio RDT, other three RDTs only marginally increased the sensitivity while combination of Reckon with any of the three RDTs was found to increase specificity to higher than 99%. However, such combined testing would double the cost. Thus, the authors concluded that Panbio IgM-based RDT alone would be a cost-effective and sensitive option especially during the times of outbreak in dengue-endemic settings [51].

The main limitation of the study is that the RDT performance was not compared with other standard tests, such as NS1 or IgM capture based ELISA or RT-PCR. There are standard ways of laboratory-based confirmation of dengue infection using various assays that are available. While the authors indicate that using CRC as case definition is commonly done, such an assessment of RDT performance may not be most accurate. Also, as acknowledged by the authors, the study results could have been affected by cross-reactivity with other flaviviruses circulating in the study area [52]. Also, dengue RDTs are commonly used especially in the areas of high incidence of dengue [56]. However, when the study measured prevalence of dengue, the authors found 15.9 to 49.3% of IgG positivity among the samples in the study and it was comparatively lower than prevalence of IgG positivity previously measured by other studies. If prevalence of dengue or other flaviviruses is lower than what was previously estimated, then performance of the RDTs would have been different in cases of low-level transmission of dengue or other flaviviruses.

Based on an economic evaluation using cost-effectiveness modeling, Lubell et al. showed that the a dengue RDT would provide little or no advantage in terms of health outcomes among patients with AFI while resulting in higher costs than current practice of antibiotics prescription [53]. As well as a dengue RDT, they had also modeled cost-effectiveness of a scrub typhus RDT and CRP test. For these two, the model showed that there are advantages over current practice of antibiotics prescription while cost would increase. There may be limited generalizability of the model outcomes, due to some of specific assumptions used in the model for this particular study sample obtained from Laos. For example, the years of life lost per death was assumed to be 45 years, based on the median age of outpatients and life expectancy in Laos. For the costs of tests, a gamma distribution was applied with a mean of $1.5, which may be lower than the current price of commonly used RDTs. The study was conducted in an outpatient-sample where dengue was confirmed in about slightly higher than 10% of the patients. While the study explored how the model outcomes would change if the incidence of dengue were to be variable between 50 and 200% of what was found in the fever study in Laos and found out that still CRP test would outperform both RDTs for dengue and scrub typhus, the study does not report how higher incidence of dengue will impact the median incremental cost and median DALYs averted by using dengue RDTs.

Also, the authors acknowledged limitations due to diagnostic uncertainty where multiple pathogens are detected for some patients whereas some others had no identifiable pathogen as the cause of illness. Depending on misclassification due to diagnostic limitations, there may be changes in economic benefit of dengue RDTs. Another limitation of the study was that the model does not consider societal impact of such viral infections where use of dengue RDTs may not be immediately cost-effective, but diagnosis based on dengue RDTs may provide benefit by raising awareness for signs of severe manifestation of illness or alerting health authorities of outbreaks for preventive and control measures, etc.

The authors, qualitatively, report that there would be improvements to current practice of antibiotics prescription whereby a dengue RDT would be used to prevent antibiotics prescribed to patients with viral infections [53]. Although not measured, there are long-term benefits of vigilant antibiotics prescription where dengue RDTs could be used for non-dengue confirmation to prompt antibiotics prescription, leading to a higher probability of bacterial infections receiving appropriate treatment. If these societal impact and long-term indirect benefits were considered in economic evaluation, dengue RDTs may be associated with higher cost-effectiveness than what was predicted in the current model.

The main assumption behind the topic of this review was the prompt detection of dengue in the early phase of illness using RDTs leading to economic impact, with both perspectives of cost-effectiveness and financial benefit. There are RDTs that detect IgA, IgM or IgG antibodies, as well as NS1 antigen [39]. Depending on the detection methods, the utility of these RDTs may be quite different and there can be variable performance characteristics. Only the study by Mitra et al. used commercially available RDTs for comparison, and Lubell et al. conducted a modeling analysis using a hypothetical RDT for dengue with 95% sensitivity and specificity in the model assumption. With limited evidence, such comparison among different test methods (or kits) could not be made in this review [42, 57]. Also, RDT performance could vary depending on factors such as the type of infection (primary vs. secondary infection), the time since onset of illness, and the serotype. It was assumed that the decision to use RDTs and refer to the test result for diagnosis and to guide clinical management would be at discretion of clinicians. Although there were no data reporting such findings, different RDTs’ variable range of performance and accuracy could lead to misclassification in terms of dengue diagnosis. And this could affect the test performance and lead to bias by under or over-estimating the economic impact of early detection of dengue. Limited by data availability and lack of assurance on the direction of bias, these factors influencing performance were not considered in this literature review.

With 2.5 billion people at risk, efforts to develop vaccine and other preventive tools continue, but dengue remains a substantial burden to the healthcare system and society in the endemic countries. [7, 58]. The total annual global cost of dengue illness was estimated at US$8·9 billion and in a large country like Brazil, it is reported that the estimated cost for dengue for the epidemic season in the societal perspective would reach as high as US$ 1212 million after adjusting for under-reporting [28, 58, 59]. In a study reviewing medical costs associated with case management for dengue fever patients in Mexico, real costs for patients, reported to the Secretariat of Health, were US$33 for outpatients, and US$491 for inpatients [60]. How burdensome dengue treatment costs would be to households was shown in a study conducted in Cambodia where survey results were compared in households with dengue positive and the ones with dengue-negative children [61]. On average, the total cost of lab-confirmed dengue was 31.5 USD and the total cost per hospitalized dengue case was 40.1 USD [61]. To finance the cost of a febrile illness, 67% of households incurred an average debt of 23.5 USD [61]. Compared to an average one-week expenditure on food in Cambodia, about 9.5 US dollars per household, the costs of treatment for dengue, whether outpatient or hospitalized, put enormous financial strain on the household [61].

Given this burden and financial strain placed by dengue on the health system, as well as individuals and households, many of the articles reviewed acknowledge the need for accurate and simple diagnostic assays for infection in resource-limited settings in regions of high dengue endemicity. However, we have found only two studies with different conclusions reached: one concluded that Panbio RDT at 6.90 USD was cost-effective; the other concluded that a dengue RDT is associated with negative DALYs averted while resulting in higher costs than current practice of antibiotics prescription. The two studies differ in design and findings cannot be directly compared. With no additional studies that explicitly estimated the cost-effectiveness of RDTs for dengue other than these two studies, such assessments must await future studies for more conclusive evidence. Likewise, any economic impact of RDT use in clinical settings, for patients, to health systems, and for particular situations such as outbreaks, remains to be assessed. Such work would guide appropriate interventions to improve patient management in resource-limited settings to reduce the burden of dengue.

## Conclusions

Existing studies of dengue RDTs are largely epidemiological and we found two studies which reported quantitative and qualitative economic impact of their use. However, these two studies reported different conclusions and there is a need for new studies to specifically measure economic impact of dengue RDTs. Such studies would yield greater understanding of the benefit of RDTs for dengue and hence could help reduce the costs incurred due to dengue illness.

## Additional file

Additional file 1:
**Table S1.** Basic information and introduction of the articles. **Table S2A.** Data extracted from the articles (study methods). **Table S2B.** Data extracted from the articles (results and discussion). (DOCX 25 kb)

## Abbreviations

AFI: Acute febrile illness
CHEERS: Consolidated Health Economic Evaluation Reporting Standards
CRP: C-reactive protein
DALYs: Disability adjusted life years
DENV: Dengue virus
ELISA: Enzyme-linked immunosorbent assay
ICER: Incremental cost-effectiveness ratios
IgM/IgG: Immunoglobulin type M and type G
NS1: Nonstructural protein 1
PRISMA: Preferred Reporting Items for Systematic Reviews and Meta-Analyses
PRNT: Plaque Reduction Neutralization Test
RDTs: rapid diagnostic tests
RT-PCR: Reverse Transcriptase-Polymerase Chain Reaction

## References

[1] Alphey N, Alphey L, Bonsall MB (2011). A model framework to estimate impact and cost of genetics-based sterile insect methods for dengue vector control. PLoS One.

[2] Clark DV, Mammen MP, Nisalak A, Puthimethee V, Endy TP (2005). Economic impact of dengue fever/dengue hemorrhagic fever in Thailand at the family and population levels. Am J Trop Med Hyg.

[3] Endy TP, Yoon IK, Mammen MP (2010). Prospective cohort studies of dengue viral transmission and severity of disease. Curr Top Microbiol Immunol.

[4] Stephenson JR (2005). The problem with dengue. Trans R Soc Trop Med Hyg.

[5] Bhatt S, Gething PW, Brady OJ, Messina JP, Farlow AW, Moyes CL, Drake JM, Brownstein JS, Hoen AG, Sankoh O (2013). The global distribution and burden of dengue. Nature.

[6] WHO: Global Strategy for Dengue Prevention and Control: 2012–2020 WHO report In*.* 2012.

[7] Brady OJ, Gething PW, Bhatt S, et al. Refining the global spatial limits of dengue virus transmission by evidence-based consensus. PLoS Negl Trop Dis. 2012;6(8):e1760. 10.1371/journal.pntd.0001760 PMC3413714 22880140

[8] Chang M, Christophel E, Gopinath D (2011). Abdur R, on behalf of malaria OVaPD, World Health Organization regional Office for the Western Pacific: challenges and future perspective for dengue vector control in the western Pacific region, regional analysis. Western Pacific Surveill Response J.

[9] Eisen L, Beaty B, Morrison A, Scott T (2009). ProactiveVector control strategies and improved monitoring and evaluation practices for dengue prevention. J Med Entomol.

[10] Villar L, Dayan G, Arredondo-García J, Rivera D, Cunha R, Deseda C, Reynales H, Costa M, Morales-Ramírez J, Carrasquilla G (2015). Efficacy of a tetravalent dengue vaccine in children in Latin America. N Engl J Med.

[11] Capeding M, Tran N, Hadinegoro S, Ismail H, Chotpitayasunondh T, Chua M, Luong C, Rusmil K, Wirawan D, Nallusamy R (2014). Clinical efficacy and safety of a novel tetravalent dengue vaccine in healthy children in Asia: a phase 3, randomised, observer-masked, placebo-controlled trial. Lancet.

[12] Larano C. Sanofi's Dengue Vaccine Made Widely Available for First Time - Philippines plans to immunize schoolchildren starting in April World 2016; http://www.wsj.com/articles/dengue-vaccine-made-widely-available-for-first-time-1456247746. Accessed May 31, 2016.

[13] Sanofi Pasteur. First Dengue Vaccine Approved in More than 10 Countries. Focus on Dengue - Press releases 2016; http://www.sanofipasteur.com/en/articles/first_dengue_vaccine_approved_in_more_than_10_countries.aspx. Accessed Oct. 31, 2017.

[14] Osorio L, Uribe M, Ardila G, Orejuela Y, Velasco M, Bonelo A, Parra B (2015). The use of rapid dengue diagnostic tests in a routine clinical setting in a dengue-endemic area of Colombia. Mem Inst Oswaldo Cruz.

[15] Blacksell SD, Bell D, Kelley J, Mammen MP, Gibbons RV, Jarman RG, Vaughn DW, Jenjaroen K, Nisalak A, Thongpaseuth S (2007). Prospective study to determine accuracy of rapid serological assays for diagnosis of acute dengue virus infection in Laos. Clin Vaccine Immunol.

[16] Peeling RW, Artsob H, Pelegrino JL, et al. Evaluation of diagnostic tests: dengue. Nature Reviews Microbiology. 2010;8:S30. 10.1038/nrmicro2459 21548185

[17] Lam SK (1994). Two decades of dengue in Malaysia. Nagasaki Univ Bull Paper Dept Tropical Med.

[18] Chen CD, Seleena B, Nazni WA, Lee HL, Masri SM, Chiang YF, Sofian-Azirun M (2006). Dengue vectors surveillance in endemic areas in Kuala Lumpur city Centre and Selangor state, Malaysia. Dengue Bulletin.

[19] da Silva LGA, Gurgel AM, Costa AM, et al. Aedes aegypti control in Brazil. The Lancet. 2016;387(10023):1052–1053. 10.1016/S0140-6736(16)00626-7 26944024

[20] Undurraga E, Betancourt-Cravioto M, Ramos-Castañeda J, Martínez-Vega R, Méndez-Galván J, Gubler D. Economic and Disease Burden of Dengue in Mexico. . PLoS Negl Trop Dis 2015;9(3):e0003547. 10.1371/journal.pntd.0003547 PMC4364886 25786225

[21] Anderson KB, Chunsuttiwat S, Nisalak A, Mammen MP, Libraty DH, Rothman AL, Green S, Vaughn DW, Ennis FA, Endy TP (2007). Burden of symptomatic dengue infection in children at primary school in Thailand: a prospective study. Lancet.

[22] Okanurak K, Sornmani S, Indaratna K (1997). The cost of dengue hemorrhagic fever in Thailand. Southeast Asian J Trop Med Public Health.

[23] Shepard DS, Undurraga EA, Halasa YA (2013). Economic and disease burden of dengue in Southeast Asia. PLoS Negl Trop Dis.

[24] Taliberti H (2010). Zucchi P: [direct costs of the dengue fever control and prevention program in 2005 in the City of Sao Paulo]. Rev Panam Salud Publica.

[25] de Araujo JM, Schatzmayr HG, de Filippis AM, Dos Santos FB, Cardoso MA, Britto C, Coelho JM, Nogueira RM (2009). A retrospective survey of dengue virus infection in fatal cases from an epidemic in Brazil. J Virol Methods.

[26] Castaneda-Orjuela C, Diaz H, Alvis-Guzman N, et al. Burden of Disease and Economic Impact of Dengue and Severe Dengue in Colombia, 2011. Value in Health Regional Issues. 1 (2) (pp 123-128), 2012. Date of Publication: December 2012.; 2012. 10.1016/j.vhri.2012.09.014 29702890

[27] Shepard D, Undurraga E, Halasa Y, Stanaway J. The global economic burden of dengue: a systematic analysis. Lancet Infect Dis. . 2016;S1473(3099(16)):146-148. 10.1016/S1473-3099(16)00146-8 27091092

[28] Martelli C, Siqueira JJ, Parente M, et al. Economic Impact of Dengue: Multicenter Study across Four Brazilian Regions. PLoS Negl Trop Dis. 2015;9(9):e0004042. 10.1371/journal.pntd.0004042 PMC4581827 26402905

[29] Constenla D, Garcia C, Lefcourt N. Assessing the Economics of Dengue: Results from a Systematic Review of the Literature and Expert Survey. Pharmacoeconomics. 2015;33(11):1107-1135. 10.1007/s40273-015-0294-7 26048354

[30] US CDC. Laboratory Guidance and Diagnostic Testing. Dengue>Clinical/Laboratory Guidance 2010. Accessed April 6, 2017.

[31] Sa-ngasang A, Wibulwattanakij S, Chanama S, O-rapinpatipat A, A-nuegoonpipat A, Anantapreecha S, Sawanpanyalert P, Kurane I (2003). Evaluation of RT-PCR as a tool for diagnosis of secondary dengue virus infection. Jpn J Infect Dis.

[32] Guzman MG, Halstead SB, Artsob H, Buchy P, Farrar J, Gubler DJ, Hunsperger E, Kroeger A, Margolis HS, Martinez E (2010). Dengue: a continuing global threat. Nat Rev Microbiol.

[33] Buchy P, Yoksan S, Peeling RW, Hunsperger E. Laboratory Tests For The Diagnosis Of Dengue Virus Infection. In: in WHOobotSPfRaT, Tropical Diseases, eds. Scientific Working Group, Report on Dengue, 1-5 October 2006, Geneva, Switzerland 2007.

[34] Thomas SJ, Nisalak A, Anderson KB, Libraty DH, Kalayanarooj S, Vaughn DW, Putnak R, Gibbons RV, Jarman R, Endy TP (2009). Dengue plaque reduction neutralization test (PRNT) in primary and secondary dengue virus infections: how alterations in assay conditions impact performance. Am J Trop Med Hyg.

[35] Putnak J, de la Barrera R, Burgess T, Pardo J, Dessy F, Gheysen D, Lobet Y, Green S, Endy T, Thomas S (2008). Comparative evaluation of three assays for measurement of dengue virus neutralizing antibodies. Am J Trop Med Hyg.

[36] Liu L, Wen K, Li J, Hu D, Huang Y, Qiu L, Cai J, Che X (2012). Comparison of plaque- and enzyme-linked immunospot-based assays to measure the neutralizing activities of monoclonal antibodies specific to domain III of dengue virus envelope protein. Clin Vaccine Immunol.

[37] Kao CL, King CC, Chao DY, Wu HL, Chang GJ (2005). Laboratory diagnosis of dengue virus infection: current and future perspectives in clinical diagnosis and public health. J Microbiol Immunol Infect.

[38] Blacksell SD. Commercial Dengue Rapid Diagnostic Tests for Point-of-Care Application: Recent Evaluations and Future Needs? Journal of Biomedicine and Biotechnology. 2012;2012(Article ID 151967):12 pages. 10.1155/2012/151967 PMC3357944 22654479

[39] Krishnananthasivam S, Fernando A, Tippalagama R, Tennekoon R, De Man J, Seneviratne D, Premawansa S, Premawansa G, De Silva A (2015). Evaluation of a commercial rapid test kit for detection of acute dengue infection. Southeast Asian J Trop Med Public Health.

[40] Hunsperger E, Sharp T, Lalita P, Tikomaidraubuta K, Cardoso Y, Naivalu T, Khan A, Marfel M, Hancock W, Tomashek K (2016). Use of a rapid test for diagnosis of dengue during suspected dengue outbreaks in resource-limited regions. J Clin Microbiol.

[41] Blacksell SD, Jarman RG, Bailey MS, Tanganuchitcharnchai A, Jenjaroen K, Gibbons RV, Paris DH, Premaratna R, de Silva HJ, Lalloo DG (2011). Evaluation of six commercial point-of-care tests for diagnosis of acute dengue infections: the need for combining NS1 antigen and IgM/IgG antibody detection to achieve acceptable levels of accuracy. Clin Vaccine Immunol.

[42] Teles FR, Prazeres DM, Lima-Filho JL (2005). Trends in dengue diagnosis. Rev Med Virol.

[43] Osorio L, Ramirez M, Bonelo A, Villar LA, Parra B (2010). Comparison of the diagnostic accuracy of commercial NS1-based diagnostic tests for early dengue infection. Virol J.

[44] Guzman MGJT, Gaczkowski R, Ty Hang VT, Sekaran SD, Kroeger A, Vazquez S, Ruiz D, Martinez E, Mercado JC, Balmaseda A, Harris E, Dimano E, Leano PS, Yoksan S, Villegas E, Benduzu H, Villalobos I, Farrar J, Simmons CP (2010). Multi-country evaluation of the sensitivity and specificity of two commercially-available NS1 ELISA assays for dengue diagnosis. PLoS Negl Trop Dis.

[45] Gan VCTL, Lye DC, Pok KY, Mok SQ, Chua RC, Leo YS, Ng LC (2014). Diagnosing dengue at the point-of-care: utility of a rapid combined diagnostic kit in Singapore. PLoS One.

[46] U.S. National Library of Medicine. Indexing with MeSH Vocabulary. PubMed Online Training 2001; https://www.nlm.nih.gov/bsd/disted/pubmedtutorial/015_030.html. Accessed 21 March, 2017..

[47] Husereau D, Drummond M, Petrou S, Carswell C, Moher D, Greenberg D, Augustovski F, Briggs AH, Mauskopf J, Loder E (2013). Consolidated health economic evaluation reporting standards (CHEERS)--explanation and elaboration: a report of the ISPOR health economic evaluation publication guidelines good reporting practices task force. Value Health.

[48] Sanders GD, Neumann PJ, Basu A, Brock DW, Feeny D, Krahn M, Kuntz KM, Meltzer DO, Owens DK, Prosser LA (2016). Recommendations for Conduct,Methodological practices, and reporting of cost-effectiveness analyses - second panel on cost-effectiveness in health and medicine. JAMA.

[49] Moher D, Liberati A, Tetzlaff J, Altman D, The PRISMA Group. Preferred Reporting Items for Systematic Reviews and Meta-Analyses: The PRISMA Statement. PLoS Medicine. 2009;6(7):e1000097. 10.1371/journal.pmed.1000097 PMC2707599 19621072

[50] Mitra S, Choudhari R, Nori H (2014). Performance and cost-effectiveness of immunochromatography based rapid diagnostic test (RDT) kits in diagnosis of dengue infection in resource limited set up. Int J Infect Dis.

[51] Mitra S, Choudhari R, Nori H (2016). Comparative evaluation of validity and cost-benefit analysis of rapid diagnostic test (RDT) kits in diagnosis of dengue infection using composite reference criteria: a cross-sectional study from south India. J Vector Borne Dis.

[52] Lubell Y, Althaus T, Blacksell SD (2016). Modelling the impact and cost-effectiveness of biomarker tests as compared with pathogen-specific diagnostics in the Management of Undifferentiated Fever in remote tropical settings. PLoS One.

[53] Ngwe Tun M, Kyaw A, Makki N, Muthugala R, Nabeshima T, Inoue S, Hayasaka D, Moi M, Buerano C, Thwe S (2016). Characterization of the 2013 dengue epidemic in Myanmar with dengue virus 1 as the dominant serotype. Infect Genet Evol.

[54] Kotaki T, Yamanaka A, Mulyatno K, Churrotin S, Sucipto T, Labiqah A, Ahwanah N, Soegijanto S, Kameoka M, Konishi E (2016). Divergence of the dengue virus type 2 cosmopolitan genotype associated with two predominant serotype shifts between 1 and 2 in Surabaya, Indonesia, 2008-2014. Infect Genet Evol.

[55] Acestor N, Cooksey R, Newton P, Menard D, Guerin P, Nakagawa J (2012). Mapping the aetiology of non-malarial febrile illness in Southeast Asia through a systematic review - terra incognita impairing treatment policies. PLoS One.

[56] Hang VT, Nguyet NM, Trung DT, Tricou V, Yoksan S, Dung NM, Van Ngoc T, Hien TT, Farrar J, Wills B (2009). Diagnostic accuracy of NS1 ELISA and lateral flow rapid tests for dengue sensitivity, specificity and relationship to viraemia and antibody responses. PLoS Negl Trop Dis.

[57] Simmons CP, Farrar JJ. Chau NvV, Wills B: Dengue. N Engl J Med. 2012; 10.1056/NEJMra1110265 22494122

[58] Shepard D, Undurraga E, Halasa Y, Stanaway J (2016). The global economic burden of dengue: a systematic analysis. Lancet Infect Dis.

[59] Lee JS, Mogasale V, Lim JKC, Mabel, et al. A Multi-country Study of the Household Willingness-to-Pay for Dengue Vaccines: Household Surveys in Vietnam, Thailand, and Colombia. PLoS Negl Trop Dis 2015;9(6). 10.1371/journal.pntd.0003810 PMC4452082 26030922

[60] Zubieta-Zavala A, Salinas-Escudero G, Ramírez-Chávez A, et al. Calculation of the Average Cost per Case of Dengue Fever in Mexico Using a Micro-Costing Approach. PLoS Negl Trop Dis. 2016;10(8):e0004897. 10.1371/journal.pntd.0004897 PMC4976855 27501146

[61] Huy R, Wichmann O, Beatty M, Ngan C, Duong S, Margolis H, Vong S (2009). Cost of dengue and other febrile illnesses to households in rural Cambodia: a prospective community-based case-control study. BMC Public Health.

