# Supplementary material for: A systematic review of the economic impact of rapid diagnostic tests for dengue
Source: BMC Health Serv Res. 2017 Dec 29;17:850. doi: 10.1186/s12913-017-2789-8 (PMC5747037; doi:10.1186/s12913-017-2789-8)
Supplement: Additional file 1: — Table S1. Basic information and introduction of the articles. Table S2A. Data extracted from the articles (study methods). Table S2B. Data extracted from the articles (results and discussion). (DOCX 25 kb) [file 12913_2017_2789_MOESM1_ESM.docx]

Supplementary File (Tables 1, 2A, and 2B)

Table 1. Basic information and introduction of the articles

| **Basic information** | | | | **Introduction** |
| --- | --- | --- | --- | --- |
| **Authors** | **Year of publication** | **Journal** | **Title** | **Context/ study question (relevant to the review question)** |
| Mitra, Shubhanker; Choudhari, Rajat; Nori, Harshita; et al. &  A meeting abstract by Mitra, S.; Choudhari, R.; Nori, H.; et al. | 2016 | JOURNAL OF VECTOR BORNE DISEASES  &  INTERNATIONAL JOURNAL OF INFECTIOUS Meeting Abstract (2014) | [Comparative evaluation of validity and cost-benefit analysis of rapid diagnostic test (RDT) kits in diagnosis of dengue infection using composite reference criteria: A cross-sectional study from south India](http://apps.webofknowledge.com/full_record.do?product=WOS&search_mode=GeneralSearch&qid=3&SID=W1f9AOpOdaWMzRDdoSa&page=1&doc=9)  Performance and cost-effectiveness of immunochromatography based rapid diagnostic test (RDT) kits in diagnosis of dengue infection in resource limited set up | To determine the sensitivity, specificity and predictive value of four commercially available RDTs [Panbio Dengue Duo cassette,  Standard Diagnostics (SD) Bioline Dengue Duo, J. Mitra Dengue Day-1 test and Reckon Dengue IgG/IgM] against composite reference criteria (CRC), and compare the cost of the tests |
| Lubell, Yoel; Althaus, Thomas; Blacksell, Stuart D.; et al. | 2016 | PLOS ONE | Modelling the impact and cost-effectiveness of biomarker tests as compared with pathogen-specific diagnostics in the management of undifferentiated fever in remote tropical settings | To assess the ability of dengue and scrub typhus rapid tests to improve antibiotic targeting in primary care, as compared with testing for elevated C-Reactive Protein (CRP), a biomarker of host inflammation  To determine the likely cost-effectiveness of the approaches as compared with current practice in community care of febrile patients in the rural tropics |

Table 2A. Data extracted from the articles (study methods)

| **Article number** | **Authors** | **Methods** | | | | | | | | | |
| --- | --- | --- | --- | --- | --- | --- | --- | --- | --- | --- | --- |
|  |  | **Target population** | **Population size** | **Age** | **location** | **Study design** | **Cost being evaluated** | **Comparators** | **Time horizon** | **Health outcome** | **# cases** |
| 1 | Mitra et al. (including the meeting abstract) | Patients who sought care for AFI at the study hospital in Vellore, India  (using stored blood samples) | 281 patients with community acquired acute  febrile illness | >18 years | Christian Medical College (CMC),  Vellore, India | prospective cross-sectional observational  study | The cost per test (as per manufacturer’s quoted price  in India) | used the composite reference criteria (CRC) for diagnosis  of dengue-related illness to compare the performance  of the RDTs from 4 manufacturers (Panbio, SD, J.Mitra and Reckon) | September 2012-February 2013 | Dengue infections against other cases of proven alternative diagnosis | 132 cases of dengue (149 controls) |
| 2 | Lubell et al. | Outpatients who sought care with fever | 1083 outpatients (among 1938 febrile patients recruited) | 5-49 years | three provincial hospitals in the provinces of Salavan, Luang Namtha, and Xieng Khouang in rural Laos | Cost-effectiveness modelling based on data from a hospital-based prospective fever study | cost effectiveness of different testing approaches, including a dengue RDT | the ability of dengue and scrub typhus rapid tests to inform antibiotic treatment,  as compared with testing for elevated C-Reactive Protein (CRP) | (based on data collected) May, 2008- December, 2010 | A viral/bacterial infection (Influenza, leptospirosis, scrub typhus, dengue, etc.) | 156 dengue cases |

| Article number | **Methods** | | | | | | | |
| --- | --- | --- | --- | --- | --- | --- | --- | --- |
|  | **Effectiveness** | | | **Preference based (measurement**  **/valuation)** | **Estimated costs (resources)** | **Currency (price date/conversion)** | **Model choice** | **Methods** |
|  | **Sensitivity & specificity** | **Single study or synthesis-based** | **Note.** |  |  |  |  |  |
| 1 | \| Manufacturer \| Comparison of the performance of dengue RDTs \| \| \| \| \| --- \| --- \| --- \| --- \| --- \| \| IgM assay \| \| NS1 assay \| \| \| Sensitivity (%) (95% CI) \| Specificity (%) (95% CI) \| Sensitivity (%) (95% CI) \| Specificity (%) (95% CI) \| \| Panbio \| 97.7 (93.5–99.5) \| 87.8 (81.5–92.5) \| NA \| NA \| \| SD Bioline \| 64.3 (55.4–72.6) \| 96.6 (92.2–98.9) \| 20.9 (14.3–28.9) \| 97.3 (93.2–99.2) \| \| Reckon \| 13.9 (8.6–21.2) \| 99.3 (96.2–99.9) \| 18.6 (12.3–26.4) \| 96.6 (92.2–98.9) \| \| J. Mitra \| 36.4 (28.1–45.4) \| 68.7 (60.6–76.1) \| 27.1 (19.7–35.7) \| 92.5 (87.0–96.2) \| | Single study | Comparison of sensitivity, specificity  and predictive values of four commercial  RDTs was done  against  CRC | measurement | Cost of the study (no costs for the RDT kits -2 manufacturers [J. Mitra and Reckon] provided  test kits for testing free of cost and other two tests were part of the routine testing in the study hospital) | US$ | Four commercially available and most commonly  used RDTs were selected for the study, from the following  manufacturers: Panbio®(Dengue Duo cassette), Standard  Diagnostics Bioline (Dengue Duo), J. Mitra (Dengue  Day-1 test), and Reckon Diagnostics (Dengue IgG/  IgM) | Measuring the performance  of the four commercially available and widely  used RDTs and comparing sensitivities and specificities against CRC |
| 2 | They assumed a sensitivity and specificity of 95% for a dengue RDT when performed on presentation and for Scrub typhus IgM RDTs (based on beta distribution). Authors have also assumed the same baseline accuracy with no cross reactivity with other rickettsial infections. | Synthesis-based | - | valuation | Data obtained from a previously conducted fever study; cost of resources for the modelling efforts | US$ | Cost-effectiveness assessment of the tests in primary care setting | Economic evaluation of diagnostics using a decision tree model and calculating the no. of DALYs averted for each strategy using probability of an antibiotic being effective for the bacterial pathogens, as well as the estimated excess duration of illness and mortality in patients that did not receive antibiotics. |

Table 2B. Data extracted from the articles (results and discussion)

| Article number | **Results** | | | | **Discussion** | | | |
| --- | --- | --- | --- | --- | --- | --- | --- | --- |
|  | **Parameters** | **Incremental costs and outcomes (cost-effectiveness)** | **Uncertainty** | **Heterogeneity** | **Study findings** | **Limitations** | **Generalizability** | **Funding source** |
| 1 | Performance (accuracy value) of the RDT kits for  diagnosis of acute dengue febrile illness (sensitivity,  specificity, PPV and NPV) and different costs of the kits | The cost per test for Panbio, SD, Reckon and J. Mitra is US$ 6.90, 4.27, 3.29 and 3.61, respectively | - | seroprevalence of IgG positivity measured in the  population using Panbio IgG RDT was lower (15.9-49.3%), compared to a previously measured 93% in a household based  survey | In dengue outbreaks, Panbio IgM capture RDT alone could be a reliable and easily available test for use in resource-limited settings; other 3 RDTs of NS1 assay may not be reliable for the diagnosis of acute dengue infection with low sensitivity. The cost per test for Panbio, SD, Reckon and J. Mitra is US$ 6.90, 4.27, 3.29 and 3.61 respectively. | Comparison of the sensitivity, specificity  and predictive values of four commercial RDTs was made against  CRC, which may not be the gold standard of dengue confirmation; comparison could not be done with standard  ELISA based NS1 or IgM capture assay; further verification of the lab results (due to possible cross-reactivity with other flaviviruses) was not done due to resource constraints | Generalizable in similar settings – where dengue prevalence as high as Vellore, India | NA |
| 2 | Sensitivity and specificity of dengue  and scrub typhus tests; Mortality rate for bacterial infections in the absence of an effective antibiotic; Years of life lost per death; Cost of RDTs; Cost of a course of antibiotics; Probability of treatment in patients with  a negative dengue or scrub typhus test result (38% and uniform distribution used)  Main assumptions and inputs:  the differences in resources use are only those related to the diagnostics and treatments; other capital and labour overheads are similar in all strategies; for the  costs of tests, a gamma distribution was applied with a mean of $1.5; the cost of a course of antibiotic is set at a mean of $0.5; all self-limiting viral infections and treated bacterial infection are associated with a week of ill health with a disability weight of 0.053; bacterial infections that do not receive an appropriate treatment are associated with 1 additional week of illness and 1% mortality rate; each of these deaths is associated with a mean loss of 45 life-years (one way sensitivity analysis between 20-60); the willingness to pay threshold was set at $1400 | Median incremental cost (CrI) $1.5 (0.5; 3.2)  Median DALYs averted (CrI) -0.006 (-0.301; 0.089) for a dengue RDT.  Dengue RDT is dominated by current practice, with higher costs and fewer numbers of DALYs averted. | A probabilistic  sensitivity analysis was done with relevant distributions, to address uncertainty in most parameters. From the cost effectiveness acceptability curve, it was shown that Dengue RDT is associated being cost effective <50% at any value of willingness-to-pay | Variable utility and accuracy of tests, subject to seasonal and spatial heterogeneity, whether used alone or in combination  Variability in terms of fever etiology, the incidence of different infections, and baseline antibiotic prescription practices | Use of dengue RDTs would lead to a reduction in antibiotics prescription for viral infections, whereas use of scrub typhus RDTs would lead to a larger proportion of bacterial infections receiving antibiotics. The CRP test performed better than dengue and scrub typhus RDTs in terms of reduction in antibiotic prescription for both viral and bacterial infections. The model showed that the dengue test offers little or no advantage over current practice (-0.006 median DALYs averted). The scrub typhus averted an average 0.031 DALYs and the CRP test averted 0.017 DALYs per fever episode.  These estimates suggest that either the scrub typhus or CRP testing is likely to be cost-effective, given uncertainty in many model parameters. | These simulations use data from a fever study in which some enrollees did not have an identifiable pathogen and some others had multiple pathogens as the cause of illness. In cases of no identifiable cause, implication of the findings is not clear. For those with multiple pathogens, a positive test result for a specific viral pathogen could mistakenly suggest that no antibiotic is required, when there is also a treatable bacterial infection. There is a need for further clinical studies about these approaches. Another limitation is that it did not account for the longer-term societal health and economic costs associated with antibiotic consumption and resistance. It would be necessary to incorporate these aspects into economic evaluation for more comprehensive assessments of the costs and benefits involved. | In cases without an identified pathogen, CRP tests may be an effective tool to guide antibiotic prescription. Overall, the model outputs will have limited generalizability to the broader population of febrile patients. | The fever study where the aetiology data  originated was funded by the WHO WPRO; the  Australian Agency for International Development, the Ministry of Foreign Affairs of Japan, and the USAID; the  Foundation for Innovative New Diagnostics (the UK Department for International  Development), National Center for Immunization and Respiratory Diseases, US CDC; and the Wellcome Trust |
